# The German Hospital, Dalston

**Published:** 1893-05-20

**Authors:** 


### THE GERMAN HOSPITAL, DALSTON.

The forty-eighth anniversary dinner in support of this institution was held last week at the Hotel Metropole, Prince Henry of Battenberg presiding. Among those present were Count Metternich, Baron de Bunsen, Count Hatzfeld, Consul-General Jordan, and others. In proposing "Prosperity to the German Hospital" Prince Henry said it gave him much pleasure to be present on such an occasion, and to be of any assistance to a hospital which had a record of such splendid work extending over nearly half a century. It was unfortunate that the funds were not sufficient to bear the increased demands upon them. The hospital, which was built forty-eight years ago exclusively for the use of Germans, was now a large and general hospital, open to all, without respect of race or language. It contains 125 beds. During the last year 1,526 patients had been admitted, and including the three dispensaries attached to the hospital, 27,690 cases had been under treatment, an increase of 298. The hospital had many generous and influential supporters, but with these increased demands, and the need of keeping up with the advance of medical science, at the end of last year there was a deficit of £500, and funds were therefore most urgently needed. The subscriptions which were subsequently announced amounted to £2,536, and included £200 from the German Emperor and £50 from the Emperor of Austria.

